# Cognitive Training Improves Sleep Quality and Cognitive Function among Older Adults with Insomnia

**DOI:** 10.1371/journal.pone.0061390

**Published:** 2013-04-05

**Authors:** Iris Haimov, Evelyn Shatil

**Affiliations:** 1 Department of Psychology and the Center for Psychobiological Research, Yezreel Academic College, Emek Yezreel, Israel; 2 CogniFit Inc., New York, New York, United States of America; Federal University of Rio de Janeiro, Brazil

## Abstract

**Study Objectives:**

To investigate the effect of an eight-week, home-based, personalized, computerized cognitive training program on sleep quality and cognitive performance among older adults with insomnia.

**Design:**

Participants (n = 51) were randomly allocated to a cognitive training group (n = 34) or to an active control group (n = 17). The participants in the cognitive training group completed an eight-week, home-based, personalized, computerized cognitive training program, while the participants in the active control group completed an eight-week, home-based program involving computerized tasks that do not engage high-level cognitive functioning. Before and after training, all participants' sleep was monitored for one week by an actigraph and their cognitive performance was evaluated.

**Setting:**

Community setting: residential sleep/performance testing facility.

**Participants:**

Fifty-one older adults with insomnia (aged 65–85).

**Interventions:**

Eight weeks of computerized cognitive training for older adults with insomnia.

**Results:**

Mixed models for repeated measures analysis showed between-group improvements for the cognitive training group on both sleep quality (sleep onset latency and sleep efficiency) and cognitive performance (avoiding distractions, working memory, visual memory, general memory and naming). Hierarchical linear regressions analysis in the cognitive training group indicated that improved visual scanning is associated with earlier advent of sleep, while improved naming is associated with the reduction in wake after sleep onset and with the reduction in number of awakenings. Likewise the results indicate that improved “avoiding distractions” is associated with an increase in the duration of sleep. Moreover, the results indicate that in the active control group cognitive decline observed in working memory is associated with an increase in the time required to fall asleep.

**Conclusions:**

New learning is instrumental in promoting initiation and maintenance of sleep in older adults with insomnia. Lasting and personalized cognitive training is particularly indicated to generate the type of learning necessary for combined cognitive and sleep enhancements in this population.

**Trial Registration:**

ClinicalTrials.gov NCT00901641

## Introduction

### Insomnia in Older Adults

Insomnia is a sleep disorder frequently observed in older persons. Its causes are varied, and in many patients there may be more than one cause. According to epidemiological data, the prevalence of chronic late-life insomnia ranges from 20% to nearly 50%, and is generally higher in women than in men [1]. Late-life insomnia is associated with changes in the architecture of sleep. Compared with younger adults, older adults spend less time in SWS and in REM sleep [2]–[4], with reductions in delta wave amplitude [3], activity and density of REM sleep and sleep spindles [2], [4]–[7]. As a result, older adults' sleep is more fragmented, with frequent and longer awakenings [3], [8], [9]. Likewise, the ability to initiate and maintain sleep declines [3], [10], along with a significant reduction in total sleep time [3], [11]–[13].

In addition to primary insomnia, insomnia in the elderly population can have medical, psychiatric, and pharmacologic etiologies [9], [14]–[16]. McCrae & Lichstein (2001) reported that co-morbid insomnia is more common and more severe in older persons compared to young adults for a variety of reasons [15]. The gradual decline of general health with age is accompanied by higher rates of medical illness. Hence, the risk of late-life insomnia is increased by the illness itself, as well as by the medication used in its treatment [17]. Likewise, older adults may also be particularly vulnerable to psychiatric co-morbid insomnia. Factors such as retirement, bereavement, social isolation and restricted movement resulting from disability occur more frequently in older adults and may affect sleep by provoking anxiety or depression [9], [14]–[16].

Moreover, since age-related changes in sleep architecture cause more fragmented sleep with lower SWS, increased stage 1, and increased awakenings, the sleep of older adults is more vulnerable to disruption by medical and psychiatric disorders compared to that of young adults [18], [19]. As a result, chronic late-life insomnia can also have a dual etiology, such that it is partially due to the primary condition and partially independent [15], [16]. Chronic late-life insomnia, independent of its underlying etiology, can have a significant negative impact on quality of life, may be a risk factor for poor health, depression, substance abuse, and mortality, and is associated with increased cardiovascular risk [20]–[23].

The most common treatment today for older adults with insomnia is pharmacotherapy, with a significant number of older people taking sleeping pills each day. However, these medications pose certain risks, such as adverse effects and dependence [9], [24], [25], and their effectiveness in insomnia wanes rapidly after 30 days of use [26]. The disadvantages of drug treatment for insomnia in older people underline the importance of non-pharmacological alternatives.

### Cognitive Performance of Older Adults with Insomnia

It is well documented that along with the changes in sleep structure accompanying the ageing process, ageing is also associated with cognitive impairment. The existing literature suggests that ageing is associated with deteriorating performance on various cognitive tasks: speed of processing information, perceptual speed, executive functioning, concentration and attention, inhibition functioning, and memory [27]. Nearly half of persons aged 60 years and older dwelling in the community express concern about declining mental abilities [27], [28], while the prevalence of mild to severe cognitive deficits in the older adult population living in the community is 4%–10% [29], [30]. Several longitudinal studies have shown an increased risk of mortality in non-demented older adult individuals with cognitive impairments [31], [32].

Recent evidence suggests that older adults with insomnia present with a pattern of cognitive deficits, over and beyond those observed in normal ageing. In a study that followed nearly 6,000 older participants over three years, the performance of older adults with insomnia decreased on tasks including balance, attention, reaction time and accessibility to information stored as semantic memory [33]. A further study comparing 24 older adults with insomnia to 50 without insomnia demonstrated that chronic insomnia in older adults is associated with impairments in episodic memory, a reduced rate of learning and temporal order judgement, and reduced resistance to proactive interference compared to good sleepers [34]. Haimov et al. (2008) reported significant differences between 35 older adults with insomnia and 64 without insomnia on tasks requiring sustained engagement of the attention system, including memory span, allocating attention to a target, time estimation and executive function [35]. Likewise, in a recently published study, 3,132 community-dwelling older men participated in research examining the association of objectively and subjectively measured sleep characteristics with cognition. They found cognition to have modest cross-sectional associations with wake after sleep onset and with self-reported long sleep [36].

The evidence for the existence of cognitive deficits reviewed above suggests that new learning or training specifically targeting cognitive function rehabilitation may be helpful in improving cognitive performance in older adults with insomnia. Cognitive training is a particularly appropriate form of such learning as it simultaneously targets cognitive function rehabilitation and provides regular, systematic and performance-adaptive learning. Recent studies have shown that, if trained, cognitive functions such as memory, attention, speed of processing and executive function, as well as distal, untrained domains such as reading and walking, can be improved through cognitive training in ageing populations [37], as well as in populations with cognitive deficits [38]–[41]. Research on primary insomnia, although limited, suggests that patients with insomnia can benefit, albeit to a lesser extent than normal sleepers, from new learning [42]–[44] and that declarative and procedural [42]–[43] memories are improved in these populations following new visual and verbal learning.

### The Relationship between Sleep and Human Cognitive Functioning

The relationship between sleep and human cognitive functioning has been investigated extensively over the past two decades in young adults. A large body of evidence supports the role of sleep in memory encoding and consolidation. Over the last decade, a multitude of molecular, cellular, systemic and behavioural findings have demonstrated the need for sleep after learning for the consolidation of memory [45]–[48]. The existing literature suggests that sleep facilitates synaptic plasticity, promotes procedural learning processes, facilitates the consolidation of declarative memories embedded in networks of previously existing associative memories, and is important for processing emotional memories [49]–[55]. Moreover, recent studies suggest that sleep is crucial for the acquisition of new memories and that the role of sleep in the consolidation of memory traces is obligatory rather than secondary [46], [56], [57].

In conjunction with studies demonstrating the role of sleep in memory encoding and consolidation, it is generally accepted that learning affects sleep architecture. A large body of research has demonstrated the beneficial effect of learning on sleep architecture. Evidence from studies in younger and older populations without insomnia suggests that sleep architecture changes as a result of learning. Following learning, young adults exhibit an increase in proportion of REM sleep [58]; an increase in number of REMs and in REM density [59], [60]; an increase in duration of Stage 2 sleep, in the number of sleep spindles and in spindle density [60]–[65]; and an increases in slow-wave activity (SWA) [66], while older adults display an increase in number of minutes in SWS and in SWS percentage [61]. Yet to the best of our knowledge, no research has examined the beneficial effects of learning on sleep structure and cognitive function among older adults suffering from insomnia.

### Research Objectives

In view of the findings showing that sleep during the night is critical in the consolidation of previously acquired memory traces, we hypothesized that the intensive new learning experience provided by systematic cognitive training will act as a catalyst to change sleep architecture and by doing so will improve sleep quality among these patients. Furthermore, we posited that if that learning specifically targets cognitive function, older people with insomnia will also exhibit improved cognitive performance.

Thus, the present study had two main objectives. The first was to examine the impact of cognitive training on sleep quality and cognitive performance among older adults with insomnia. The second was to examine the impact of cognitive training on the relation between the changes in cognitive function and those in sleep quality among these patients.

Consequently, the present study seeks to verify whether cognitive training may be used as a novel non-pharmacological alternative to improve the sleep quality of older adults suffering from insomnia. The present study constitutes pioneering work in this field among older adults with insomnia.

## Methods

The protocol for this trial and supporting TREND checklist are available as supporting information; see Checklist S1 and Protocol S1.

### Study Design

**Figure 1:**
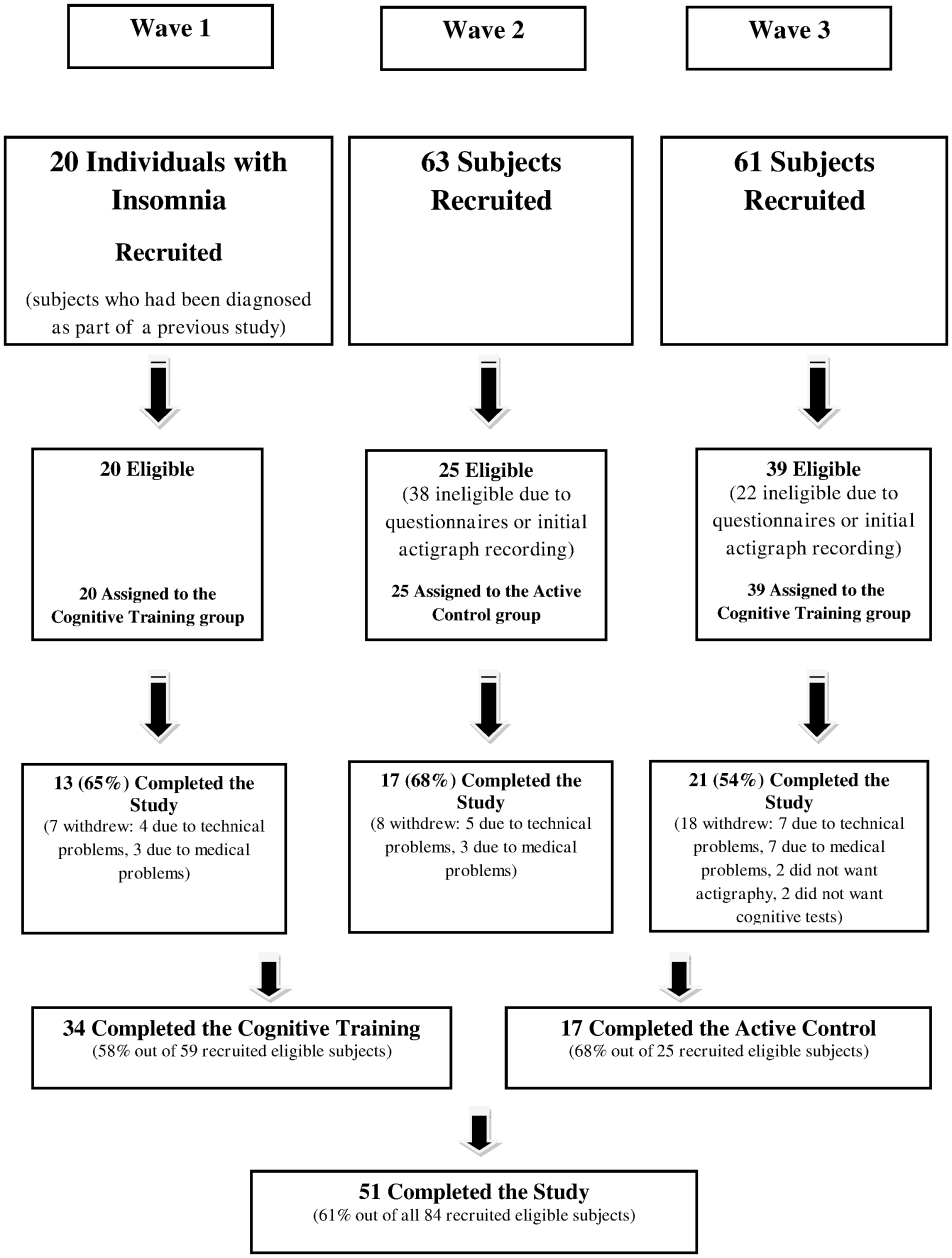
Participant Flowchart.

A randomized controlled, proof of concept, eleven-week long, clinical trial evaluated the efficacy of cognitive remediation on improving quality of sleep in older adults living independently in the community and diagnosed with chronic insomnia (AASM criteria), using a two-group design: intervention and active comparator control. To guarantee full blindness recruitment was planned in four waves, with the goal of reaching a total of 68 study completers, 34 in each study group. The first recruited participants were assigned to the cognitive training group, the second to the active control group, and the third to the cognitive training group. The fourth study wave, designed to recruit new control subjects was not implemented as results obtained from data collected in the first three waves were deemed satisfactory. Figure 1 describes recruitment and adherence patterns for the implemented first three waves.

### Eligibility

Potential participants were contacted through advertisements and talks given at local senior centres. Applicants were asked to complete several questionnaires. Exclusion criteria included a score <26 on the mini-mental state examination (MMSE) [67], a score >40 on the Zung self-rating depression scale (ZSDS) [68] and a score >60 on the short anxiety questionnaire [69]. Additional exclusion criteria included: (a) significant visual or hearing impairments; (b) significant medical or neurological disease, including active cancer (ongoing chemotherapy or other cancer treatment), diabetes, liver, kidney, heart or lung disease; (c) alcoholism or other drug abuse or dependence; (d) history of significant psychiatric impairment such as major depression or psychosis; (e) sleep apnea; (f) periodic limb movement disorder and (g) use of centrally active medications, excepting sedatives or hypnotics prescribed for sleep.

Participants were eligible for inclusion in the current study according to AASM diagnosed criteria of chronic insomnia in adults [70], [71]. Applicants were asked to complete the Technion Sleep Questionnaire [72], which consists of 72 items on sleep habits, sleep disorders, and general health. Based on the sleep questionnaire participants were eligible for inclusion if they reported difficulties in initiating or maintaining sleep at least three nights per week and poor sleep that lasted for a minimum of six months. In addition, participants had to report daytime impairment complaints. Participants also had to report that their poor sleep was not caused by chronic pain or by any known medical disease, and that they did not use alcohol or psychiatric medication.

For objective confirmation of the participants' self-reports, the sleep of each participant was continuously monitored over a one-week period by a miniature actigraph worn on the wrist (Mini Motionlogger, Ambulatory Monitoring, Inc. Ardsley, NY), enabling monitoring of sleep under natural circumstances with minimal distortions. Participants were included in the study if during the evaluation period they exhibited (a) sleep onset latency or wake time after sleep onset of ≥31 min [73] and less than 85% sleep efficiency (percentage of total sleep time out of total time in bed) for at least three out of seven nights [74]; and (b) sleep efficiency of less than 85% when averaged across seven consecutive nights [74].

All participants owned and were able to use a personal computer, spoke fluent Hebrew, and had healthy dominant-hand functioning. The clinical experiment conformed to the principles outlined by the Declaration of Helsinki and the complete study protocol was approved by the institutional ethics committee of the Max Stern Academic College of Emek Yezreel. After the study was completely described to all participants, their written informed consent was obtained. Study par­ticipants did not receive any monetary compensation.

### The Interventions

Participants in the cognitive training group completed a home-based, personalized, computerized cognitive training program (using the CogniFit® cognitive training program). Participants in the active control group completed a home-based program involving computerized tasks that do not engage high-level cognitive functioning (“Word and Paint”). Both programs were similar in time commitment of 20–30 minutes per session and both regimens were similarly structured - three sessions each week (with a no-training day between sessions), for a duration of 8 weeks (24 training sessions). At the beginning of the study all participants completed a broad spectrum of questionnaires. In the two weeks immediately before the onset of the intervention and following the end of the intervention, baseline and post-training sleep quality data were collected i.e., during these two weeks participants' sleep was continuously monitored by actigraph and participants filled a daily sleep diary. In addition, before the onset of the intervention and following the end of the intervention participants' cognitive performance was evaluated using the CogniFit® computerized neurocognitive evaluation. A research assistant visited each participant’s home and helped install the programs (the CogniFit® computerized neurocognitive evaluation and the cognitive training program) on the participant’s home computer and provided guidelines as to the frequency and duration of training. To monitor adherence, participants were required to keep a record of the training session number, duration and date, and received a telephone call every two weeks inquiring about their progress. The different study phases and the measures collected in each phase are depicted in Table 1, while the number of visits to the participants' homes and the content of these visits are presented in Table 2.

**Table 1:** Summary of Measures Taken in Each Phase of the Study.

Baseline monitoring	Training phase (eight weeks)	Post-training monitoring
Standard clinical historyQuestionnaireTechnion SleepQuestionnaireZung self-ratingdepression scaleAnxiety questionnaireMini-Mental StateExamination(MMSE)Actigraph recordingdaily sleep diaryCogniFit®computerizedneurocognitiveevaluation	Personalized-computer-cognitive- program (CogniFit® cognitive training program)/”Word and paint”training package20 to 30 minutes, three times a week, for 8 weeks(24 training sessions).	Actigraph recordingdaily sleep diaryCogniFit® computerized neurocognitive evaluation

**Table 2:** Number of Visits to the Participants' Homes and Purpose of Each Visit.

Visit 1	Visit 2	Visit 3	Visit 4	Visit 5	Visit 6
Participants were asked to fill :**•** Clinical history questionnaire**•** Technion Sleep Questionnaire**•** Zung self-rating depression scale**•** Short anxiety questionnaire	Participants were instructed to wearthe actigraph on thewrist for seven consecutive nightsand to report eachday in the dailysleep diary.	Participants were instructed tocomplete the CogniFit® computerized neurocognitive evaluation	The computerized individuallyadaptive cognitive training program (CogniFit® cognitive training program)for the cognitive traininggroup and the computerized active comparator program(‘Word and Paint’ training package) for the activecontrol group were installedin the participants' homes on their personal computers.On this visit, the research assistant made sure that the subject could continue using the prescribed training program autonomously during the length of the program.	Participants were instructed to complete the CogniFit® computerized neurocognitive evaluation and to wear the actigraph on thewrist for seven consecutivenights and to report eachday in the daily sleep diary.	At the end of the study presentation of the results with a description of their individual actigraph results before and after training were carried out and the participants were rewarded with a complimentary cognitive training program.

#### i. The cognitive training group

During the eight-week experimental period the participants in the cognitive training group completed an eight-week, home-based, personalized, computerized cognitive training program using the CogniFit® cognitive training program. CogniFit® cognitive training program is a computer-based personalized cognitive training program that has been validated in several populations [38], [39], [75], [76], [77]. It begins with a baseline cognitive evaluation, the CogniFit® computerized neurocognitive evaluation, the results of which determine the individual level of subsequent training for each participant. Personalization is accomplished by incorporating an adaptive feature that continually measures the performance of each participant, adapts the difficulty level of the training tasks, and provides detailed graphic and verbal performance feedback after each training task. Because the program is adapted to each person’s strengths and weaknesses, it is unlikely that two participants will receive the same training regimen. The CogniFit® training program for this study consisted of 21 different training tasks, each with three levels of difficulty (easy, moderate and difficult). The level of challenge is readjusted after each training session in accordance with the participant’s progress on the tasks. A list of the training tasks and the abilities they train is included in Appendix S1.

Automatically generated weekly adherence reports were forwarded to the research coordinator. After completion of the program, the participants in the cognitive training group were administered the CogniFit® computerized neurocognitive evaluation for the second time.

#### ii. The active control group

Participants assigned to the active control group received a software program (“Word and Paint”). Their particular program did not train specific mental functions, was not adapted to participants’ performance, and did not provide any feedback. It included twelve assignments in Microsoft Word and ten in Microsoft Paint requiring participants to read poetic, narrative and expository texts, to copy the texts and manipulate font and format as well as to draw and colour pictures. Assignments were saved in a computer directory that was examined at the end of the study. After completion of the program, the participants in the active control group were administered the CogniFit® computerized neurocognitive evaluation for the second time.

### Primary Outcome Measures

Sleep quality was measured before and after training. The initial week-long actigraph monitoring, conducted to confirm participants' reports of insomnia, was used as an exclusion criteria (for those participants that failed to display insomnia) and as baseline sleep quality measures for participants included in the study. Following the eight-week training period, participants’ sleep was again similarly monitored for one week.

#### Actigraphy

In both the pre-training and the post-training sleep monitoring, participants were instructed to wear a miniature actigraph (Mini Motionlogger, Ambulatory Monitoring Inc., Ardsley, New York, USA) on their wrist for seven consecutive nights, and to press a button on it when they started trying to fall asleep and when they woke up the following morning. The first button press was used to determine bedtime and the second was used to determine wake time.

In order to precisely analyze the actigraph data, over the course of actigraphic recording subjects were given daily sleep diaries. Subjects were instructed to report the time they got into bed, when they started trying to fall asleep, when they actually fell asleep, when they woke up in the morning, when they got out of bed, and their estimate of the amount of sleep they got that night.

The actigraph enables monitoring of sleep under natural circumstances with minimal distortions. The actigraph measures wrist activity utilizing a piezoelectric element, and translates wrist movements into an electrical signal that is digitized and stored in the actigraph’s memory. The actigraph collected data in 1-min epochs (activity level was sampled at 10-sec intervals and summed across 1-minute intervals) and stored at amplifier setting 18 (i.e., manufacturer's technical code for frequency band pass 2 to 3 Hz, high gain and high threshold). This working mode is the standard mode for sleep-wake scoring [78]–[85]. Actigraphic raw data were translated to sleep measures using the Actigraphic Scoring Analysis program for an IBM-compatible personal computer (W2 scoring algorithm) provided by the manufacturer.

Actigraphy has been well validated against polysomnography in trials with people without insomnia with agreement rates for minute-by-minute sleep-wake identification of over 90% [86] [87] as well as with persons with insomnia [88]–[90]. Actigraphic sleep measures included five measures of sleep quality: total sleep time (total number of minutes defined as sleep from bedtime to wake time), sleep onset latency (time to fall asleep from bedtime), sleep efficiency (percentage of total sleep time out of total time in bed), wake time after sleep onset (total number of wake minutes after sleep onset), and number of awakenings (during sleep).

### Secondary Outcome Measures

The CogniFit® computerized neurocognitive evaluation was administered both at baseline and following training. This cognitive evaluation consists of three 20-minute sessions that measure a wide variety of cognitive abilities. Scores on 17 abilities are assigned using weights previously derived from a factor analysis performed on normative data from a healthy population. The CogniFit® computerized neurocognitive evaluation has been validated in a younger population (mean age 23 years) against several major standard neuropsychological tests, including the full Cambridge Neuropsychological Test Automated Battery, Raven’s Standard Progressive Matrices, the Wisconsin Card Sorting Test, the Continuous Performance Test, the STROOP test, and other reading tests [35].

### Analyses

SPSS 19 [91] software was used for statistical analyses. Mixed effects models for repeated measures were used to evaluate differences in the five sleep variables and in the 16 cognitive variables within and between groups; a separate model being established for each variable. The models allowed us to assess differences in baseline scores between the two groups, differences between baseline and post-training scores within each group, and whether any of the differences varied between the groups. The independent variables included group (cognitive training or active control), time (baseline or post-training), group by time interaction, and age; the dependent variable was the sleep variable or the cognitive variable. Group and time were categorical fixed factors, with the participant being the random factor. To determine whether an association exists between improvements in cognitive function and improvements in sleep quality, we calculated Pearson-moment correlations between the sleep improvements and the cognitive improvements and we conducted hierarchical regression analyses with cognitive improvements as the independent variables and the sleep improvements as the dependent variables.

## Results

### Adherence and Personal Information

144 applicants, older adults living independently in the community and with a complaint of insomnia, were recruited from several senior citizens local day centres. Based on the questionnaire and actigraphic evaluations 84 applicants, diagnosed with chronic insomnia (AASM criteria) were deemed eligible for inclusion in the current study. Of those, fifty-one participants, 34 in the cognitive training group and 17 in the active control group, completed the study. Completion rate was almost the same for both groups (58% for the cognitive training group and 68% for the active control group). Figure 1 presents adherence patterns and Table 3 shows that baseline characteristics were equivalent between the two groups of completers, with the exception of age, with the control group about three years younger on average (p<0.02). Therefore, all ensuing mixed models analyses controlled for age.

**Table 3:** Baseline Characteristics of Participants.

	Cognitive Training Group (N = 34)	Active Control Group (N = 17)	pValue
Age in years: mean (SD) [range]	73.2 (5.7) [65]–[85]	69.9 (3.9) [65]–[79]	0.02
Female (%)	53	67	0.55
Higher education (%)	42	47	0.53
Mother language Hebrew (%)	47	65	0.37
Years in retirement: mean (SD)	7.1 (6.4)	5.8 (7.1)	0.50
Working hours per day: (mean ± SD)			
Before retirement	7.8 (2.1)	7.6 (2.6)	0.73
After retirement	4.3 (0.9)	5.1 (2.1)	0.30
Family status (% married)	35	18	0.33
Psychological status: mean (SD)			
Zung Depression Scale	19.4 (4.6)	19.3 (3.6)	0.96
Short anxiety questionnaire	45.4 (10.5)	47.5 (8.9)	0.49
Using sleeping pills (%)	31	21	0.31

### Primary Outcome: Sleep Quality

Table 4 presents mixed models statistics on participants' sleep quality before and after training. Comparisons of the groups showed that sleep parameters were similar between the groups at baseline, with no significant differences (columns 10 and 11). The between-groups comparisons (columns 12) revealed that, when compared to the active control group, after controlling for age, the cognitive training group showed significant improvements on two sleep parameters: sleep onset latency and sleep efficiency. Using the Mean Differences and their Standard Deviations, Cohen‘s *d* were calculated (column 13) to examine effect size [92]. Cohen-d effect size sets a benchmark of 0.20 as small, 0.50 as medium and 0.80 as large [93]. Our results show that effects for sleep onset latency and sleep efficiency fell in the medium range (column 13). We also observed a significant effect of cognitive training within the cognitive training group for sleep onset latency, sleep efficiency, wake after sleep onset, number of awakenings (column 5) but not for total sleep time. Within the active control group (column 9) no significant effects were observed for any of the sleep variables.

**Table 4:** Mixed models statistics for within-group, baseline and between-group differences on sleep quality parameters for the two study groups after adjusting for age.

	Baseline, Post-Training scores, Standard Deviations and Within-Group Mean Differences at the End of the Intervention								Baseline Mean Differences at the Onset of the Intervention		Between-Group Mean Differences at the End of the Intervention	
	Cognitive Training Group N = 34				Active Control Group N = 17							
	BaselineMean (SD)	Post-TrainingMean (SD)	Mean Difference	F (df = 1,51)	BaselineMean (SD)	Post-TrainingMean (SD)	Mean Difference	F (df = 1,51)	Mean difference after adjusting for age	F (df)	F (df = 1,51)	Cohen's d
SOL	38.42 (40.58)	24.76 (32.32)	−13.66	12.65***	31.65 (26.36)	33.56 (23.85)	5.18	0.12	−10.40	1.09 (1, 64)	5.49*	−0.70∧∧
SE	73.54 (12.56)	80.28 (13.78)	6.74	18.22***	77.19 (12.22)	76.76 (14.04)	−0.42	0.36	4.10	1.05 (1, 64)	6.86*	0.70∧∧
TST	296.37 (78.07)	310.44 (72.96)	14.07	2.21	333.79 (70.05)	321.64 (76.4)	−12.15	0.84	42.53	3.56 (1, 66)	2.56	0.50∧
WASO	72.06 (40.89)	58.89 (45.13)	−13.17	5.05*	69.04 (36.62)	67.5 (44.91)	−1.53	0.03	−1.19	0.01 (1, 69)	0.13	−0.31∧
NA	10.65 (4.38)	9.04 (5.90)	−1.61	5.36*	10.82 (4.21)	10.5 (3.70)	−0.32	0.10	−0.05	0.001 (1, 71)	1.15	−0.32∧
Col. 1	Col. 2	Col. 3	Col. 4	Col. 5	Col. 6	Col. 7	Col. 8	Col. 9	Col. 10	Col.11	Col. 12	Col. 13

1 SOL = Sleep Onset Latency (minutes); SE = Sleep Efficiency (%); Total Sleep Time (minutes); WASO = Wake After Sleep Onset (minutes); NA = Number of Awakenings.

2 Significance levels:

* = significant at the level of 0.05,

** = significant at the level of 0.01,

*** = significant at the level of 0.001.

3 Cohen’s d effect sizes:

∧ = small-sized effect,

∧∧ = medium-sized effect,

∧∧∧ = large-sized effect.

### Secondary Outcome: Cognitive Performance

Although 51 participants completed the entire study, due to technical difficulties all cognitive performance data (from baseline and post-training) were unavailable for analysis for 6 participants (5 participants in the cognitive training group and 1 in the control group) and post-intervention data were unavailable for 11 additional subjects (10 participants in the cognitive training group and 1 in the control group). Table 5 presents mixed models statistics for the 16 cognitive abilities for the 45 participants (29 participants in the cognitive training group and 16 in the control group) who had complete or partial data. Table 5 (columns 10 and 11) show that the groups were unequal at baseline on several cognitive functions; however, these baseline differences were controlled for using the mixed models procedure used for the between-groups differences. The between-groups comparisons (columns 13) revealed that, when compared to the active control group, after controlling for age, the cognitive training group showed significant improvements on five cognitive measures: avoiding distractions, naming; general memory, visual memory and working memory. Using the Mean Differences and their Standard Deviations, Cohen’s *d* calculated for those five cognitive abilities, fell in the medium to high range (column 14). A significant effect of cognitive training was observed within the cognitive training group (column 5) for auditory (non-linguistic) memory, divided attention, naming, visual perception and visual scanning at the uncorrected alpha level of 0.05; response time and working memory at the uncorrected alpha level of 0.01; and general memory, time estimation, visual memory at the corrected alpha level of 0–.003. Within the active control group (column 9) there was a significant effect, at the corrected alpha level of 0.003 for avoiding distractions. Of special interest, in this group a significant reduction, at the uncorrected alpha level of 0.05 in mean scores was observed on working memory, a measure which had improved considerably in the cognitive training group.

**Table 5:** Mixed models statistics for within-group, baseline and between-group differences on cognitive abilities for the two study groups after adjusting for age.

	Baseline, Post-Training scores, Standard Deviations and Within-Group Mean Differences at the end of the intervention								Baseline mean differencesat the onset of theintervention		Between-Group meandifferences at the endof the intervention		
	Cognitive Training Group				Active Control Group								
	BaselineMean N = 29	Post-TrainingMean N = 20	MeanDifference	F (df)	Baseline Mean N = 16	Post-Training Mean N = 15	Mean Difference	F (df)	Mean Difference	F (df)	Mean Difference	F (df)	Cohen's d
AM	−.11(.71) N = 29	.22(.58) N = 20	.36	5.91* (1, 39)	.14(.60)	.22(.80)	.06	.10 (1, 34.8)	.27	1.59 (1, 66)	.30	1.72 (1,37)	0.5∧∧
DA	−.40(.62) N = 29	−.20(.46) N = 20	.21	4.22* (1, 39)	−.30(.44)	−.22(.70)	.04	.09 (1, 35)	.02	.01 (1, 60)	.17	1.19 (1,37)	0.5∧∧
DS	−.50(.44) N = 29	−.42(.46) N = 20	.04	1.26 (1, 36)	−.78(.20)	−.60(.27)	.17	15.74*** (1, 37)	−.31	7.02* (1, 49)	.13	5.18* (1,36)	−0.8∧∧∧
GC	−.45(.73) N = 29	−.28(.56) N = 20	.16	2.45 (1, 36)	.66(.62)	.63(.56)	−.01	.01 (1, 34)	1.09	27.38*** (1, 53)	.15	1.22 (1, 34)	0.4∧
GM	−.77(.86) N = 29	−.15(.81) N = 20	.74	15.63*** (1, 37)	.07(.79)	−.31(1.36)	−.41	3.40 (1, 33)	.74	6.07* (1, 60)	1.15	15.65*** (1, 35)	1.4∧∧∧
IN	−.10(.52) N = 29	−.14(.41) N = 20	−.02	.03 (1, 39)	−.12(.42)	.14(.68)	.26	2.80 (1,33)	.05	.12 (1, 74)	−.28	1.95 (1,35)	−0.4∧
NM	−.31(.76) N = 29	.04(.53) N = 20	.32	5.37* (1, 34)	.42(.58)	.13(.76)	−.33	3.99 (1, 30)	.68	9.86** (1, 59)	.65	9.65** (1, 32)	1.0∧∧∧
PL	−.39(1.26) N = 28	−.26(1.22) N = 20	.00	.00 (1, 35)	.02(.96)	.25(.82)	.24	.87 (1, 32)	.25	.51 (1, 59)	−.24	.49 (1,33)	−0.3∧
RT	−.98(1.46) N = 28	−.64(.89) N = 20	.47	7.70** (1, 37)	.33(.47)	.42(.42)	.06	.09 (1, 36)	1.22	14.42*** (1, 55)	.41	2.46 (1, 36)	0.8∧∧∧
SH	−.26(1.33) N = 28	−.08(.66) N = 20	.16	.60 (1, 39)	.17(.50)	.20(.73)	.03	.02 (1, 35)	.44	2.21 (1, 65)	.13	.16 (1, 37)	0.1∧∧∧
SP	−.87(2.01) N = 28	−.73(1.17) N = 20	.24	.69 (1, 41)	.31(.35)	.46(.36)	.14	.18 (1, 38)	1.61	7.38** (1, 64)	.10	.04 (1, 39)	0.2∧
TE	−.17(.98) N = 28	.35(.67) N = 20	.54	12.00*** (1, 39)	.17(.72)	.27(.75)	.09	.24 (1, 36)	.25	1.00 (1, 60)	.45	3.36 (1, 39)	0.7∧∧
VM	−.82(.83) N = 28	−.20(.73) N = 20	.73	17.05*** (1, 37)	.01(.85)	−.26(1.34)	−.29	2.00 (1, 34)	.71	6.07* (1, 58)	1.02	14.03*** (1, 35)	1.3∧∧∧
VP	−.80(1.15) N = 28	−.58(.80) N = 20	.26	5.82* (1, 35)	.29(.62)	.48(.39)	.12	.91 (1, 34)	.98	12.23*** (1, 48)	.14	.72 (1, 34)	0.4∧
VS	−99(1.59) N = 28	−.45(1.14) N = 20	.51	4.37* (1, 36)	−.16(1.31)	.37(1.17)	.54	3.52 (1, 36)	.65	2.41 (1, 58)	−.03	.01 (1, 37)	0
WM	−.75(.89) N = 28	−.21(.85) N = 20	.62	9.47** (1, 38)	.09(.69)	−.44(1.31)	−.55	5.24* (1, 34)	.71	6.01* (1, 64)	1.17	13.92*** (1, 35)	1.372∧∧∧
Col.1	Col. 2	Col.3	Col.4	Col.5	Col.6	Col.7	Col.8	Col.9	Col.10	Col 11	Col.12 Col.13		Col.14

1 AM = Auditory (non-linguistic) memory; DA = Divided Attention; DS = Avoiding Distractions; GC = Hand-eye co-ordination.

GM = General Memory; IN = Inhibition; NM = Naming; PL = Planning; RT = Response Time; SH = Shifting; SP = Spatial Perception; TE = Time estimation; VM = Visual Working Memory; VP = Visual Perception; VS = Visual Scanning; WM = Working Linguistic-Auditory Memory.

2 Significance levels:

* = significant at the level of 0.05,

** = significant at the level of 0.01,

*** = significant at the level of 0.001.

3 Cohen's d effect sizes:

∧ = small-sized effect,

∧∧ = medium-sized effect,

∧∧∧ = large-sized effect.

### Are the Sleep Improvements Associated with the Cognitive Improvements?

#### 1. Correlations between the sleep quality parameters and the cognitive function parameters

To answer this question we correlated the five sleep mean-differences (post-intervention mean minus baseline mean) with the 16 cognitive mean-differences twice, once for each group. These correlations, presented in Table 6, show that for subjects in the cognitive training group the mean differences in sleep efficiency, wake after sleep onset and number of awakenings were significantly correlated with naming ability. The mean difference in total sleep time was significantly correlated with avoiding distractions, and the mean difference in sleep onset latency was significantly correlated with visual scanning. For subjects in the control group visual memory, general memory and working memory were related to sleep onset latency. The relation in this case was a negative one: the memory measures, in this group, declined significantly (see table 5) and the negative correlation signs (see Table 6) indicate that the steeper the memory decline, the longer it took participants in this group to fall asleep. The mean-differences for the other cognitive abilities measured exhibit no relation to any of the mean-differences in the sleep parameters. We computed Pearson correlation coefficients among the three cognitive memory improvements (general memory, visual memory and working memory) and found these were highly inter-correlated (r = .955 to r = .991; p<.001). This redundancy is explained by the fact that these three cognitive abilities share some of the same constructs [39]. Working memory involves the manipulation of information stored in short-term memory while simultaneously performing a task. In our battery it is assessed by a visual-spatial backward memory task. The other two memory measures are a blend of variables that include the working memory variables but also other variables borrowed from additional memory storage and retrieval tasks. To eliminate the redundancy, we decided to analyze the working memory measure only, as it is the most specifically defined in terms of the variables that served to compute its scores. In addition, to ensure we were, indeed, selecting the most appropriate memory measure to explore the relation between the sleep and cognition improvements, we conducted a stepwise regression with the three correlated memory measures as independent variables and sleep onset latency as the dependent variable. Working memory was the only variable to enter the regression (F = 10.21, p = 0.003; b = −11.38, t = 3.20, p = 0.003). Therefore, in the ensuing regression analyses, working memory alone was used.

**Table 6:** Table **6.** Correlations between mean differences in sleep quality parameters and mean differences in cognitive abilities in the cognitive training and active control groups.

	d- Sleep Onset Latency		d_Sleep Efficiency		d_Total Sleep Time		d_Wake After Sleep Onset		d_Number of Awakenings	
	Cognitive Training	Control	Cognitive Training	Control	CognitiveTraining	Control	CognitiveTraining	Control	CognitiveTraining	Control
d_AM	.29	−.11	.22	.03	.06	.01	−.34	.18	−.14	.17
d_DA	.09	−.11	.26	.16	.37	.23	−.22	−.05	−.03	.02
d_DS	−.07	.23	.27	.10	.49*	.10	−.06	−.27	−.03	.07
d_GC	.01	.08	.08	−.15	.36	−.22	−.03	.03	.19	.11
d_IN	−.33	−.08	−.01	.31	.07	.21	.31	−.25	−.05	−.01
d_NM	.14	−.04	.32”	.19	.18	.20	−.42”	−.17	−.47*	−.01
d_PL	−.19	.15	.28	.08	.30	.08	−.16	.01	.01	.40
d_RT	.34	−.05	−.15	−.16	.03	−.03	−.11	.22	−.17	.12
d_SH	.01	.36	.00	−.23	−.02	−.21	−.07	−.07	−.10	.04
d_SP	.35	.23	−.01	−.41	.10	−.32	−.23	.39	−.21	.34
d_TE	.15	.29	−.12	−.43	−.03	−.41	.04	.29	−.08	.22
d_VP	.23	.16	−.20	.00	−.09	−.08	−.04	−.11	−.22	.17
d_WM	−.28	−.64*	−.18	.31	.01	.39	.12	−.08	.29	−.39
d_VS	.40”	.17	−.14	−.17	−.06	−.12	.02	.16	−.15	.21
d_VM	−.18	−.53*	−.14	.33	.10	.41	.11	−.17	.31	−.36
d_GM	−.23	−.58*	−.14	.32	.14	.41	.14	−.12	.33	−.39

1 d = mean-differences (post-intervention mean minus baseline mean); AM = Auditory (non-linguistic) memory; DA = Divided Attention; DS = Avoiding Distractions; GC = Hand-eye co-ordination; GM = General Memory; IN = Inhibition; NM = Naming; PL = Planning; RT = Response Time; SH = Shifting; SP = Spatial Perception; TE = Time estimation; VM = Visual Working Memory; VP = Visual Perception; VS = Visual Scanning; WM = Working Linguistic-Auditory Memory.

2 Significance levels:

“ = significant at the level of.09,

* = significant at the level of.05,

** = significant at the level of.01.

#### 2. Hierarchical regressions

**Figure 2:**
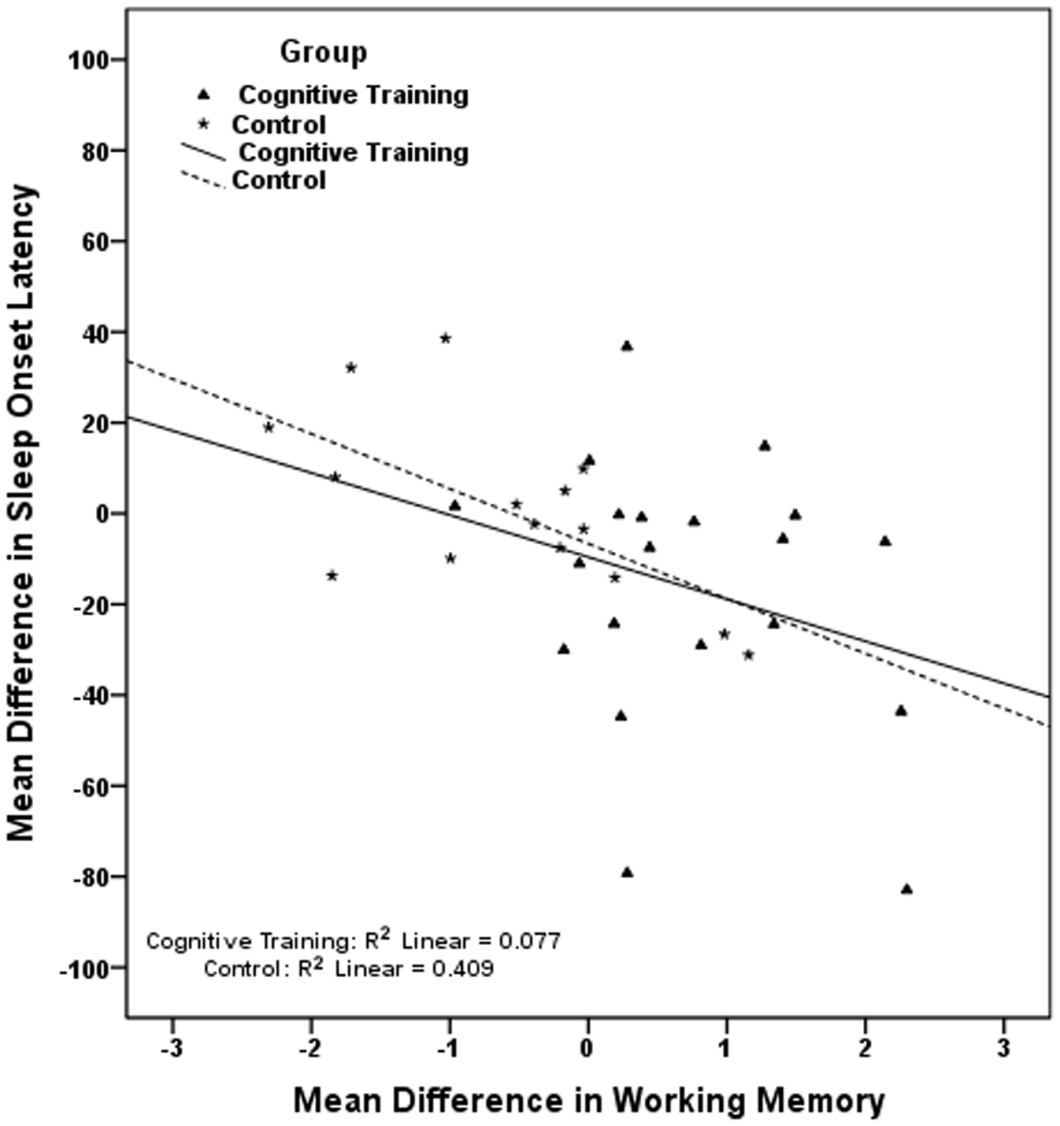
Linear regression between Mean Difference (post-intervention mean minus baseline mean) in Sleep Onset Latency (dependent) and Mean Difference (post-intervention mean minus baseline mean) in Working Memory (independent).

**Figure 3:**
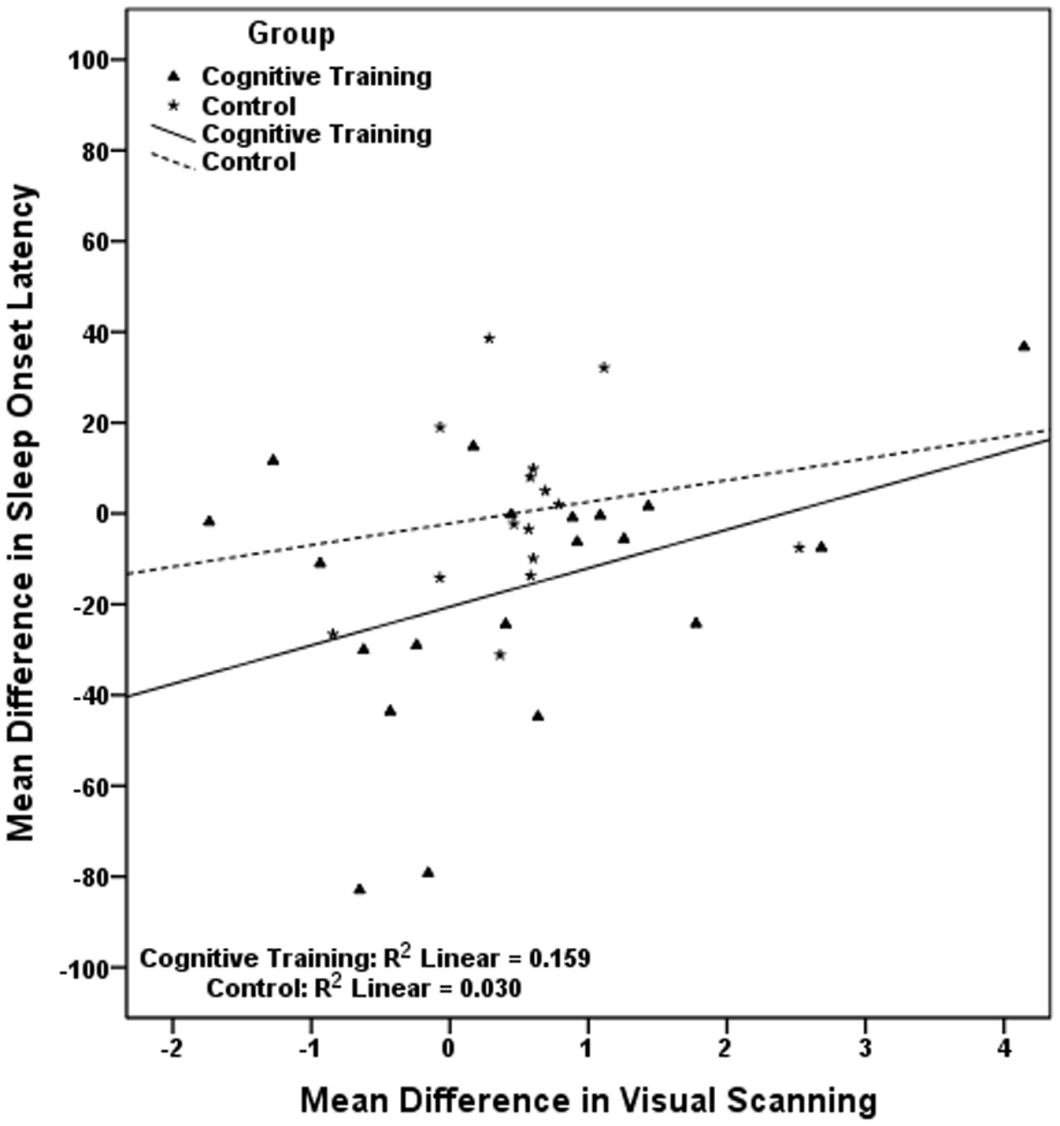
Linear regression between Mean Difference (post-intervention mean minus baseline mean) in Sleep Onset Latency (dependent) and Mean Difference (post-intervention mean minus baseline mean) in Visual Scanning (independent).

**Figure 4:**
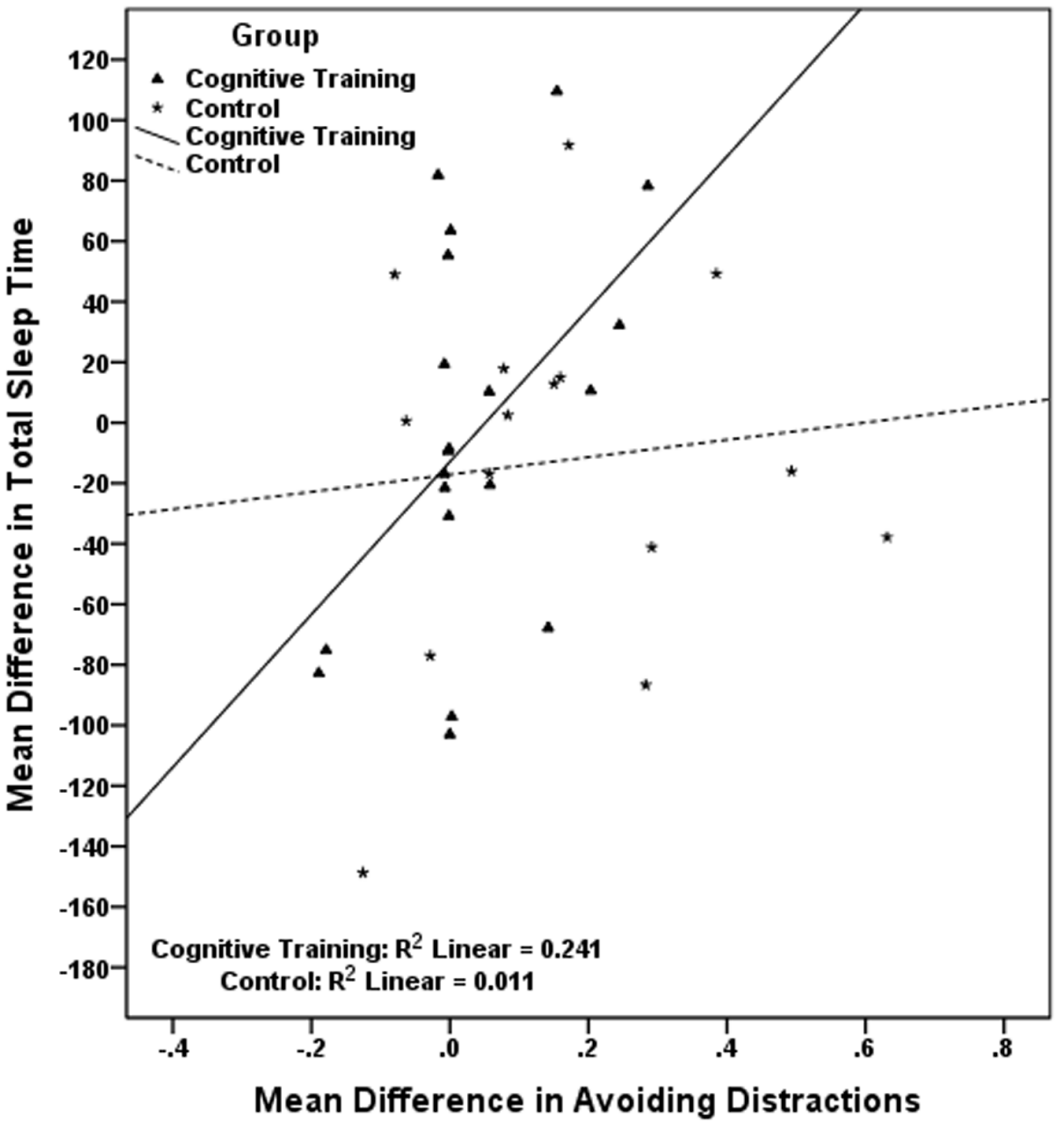
Linear regression between Mean Difference (post-intervention mean minus baseline mean) in Total Sleep Time (dependent) and Mean Difference (post-intervention mean minus baseline mean) in Avoiding Distractions (independent).

**Figure 5:**
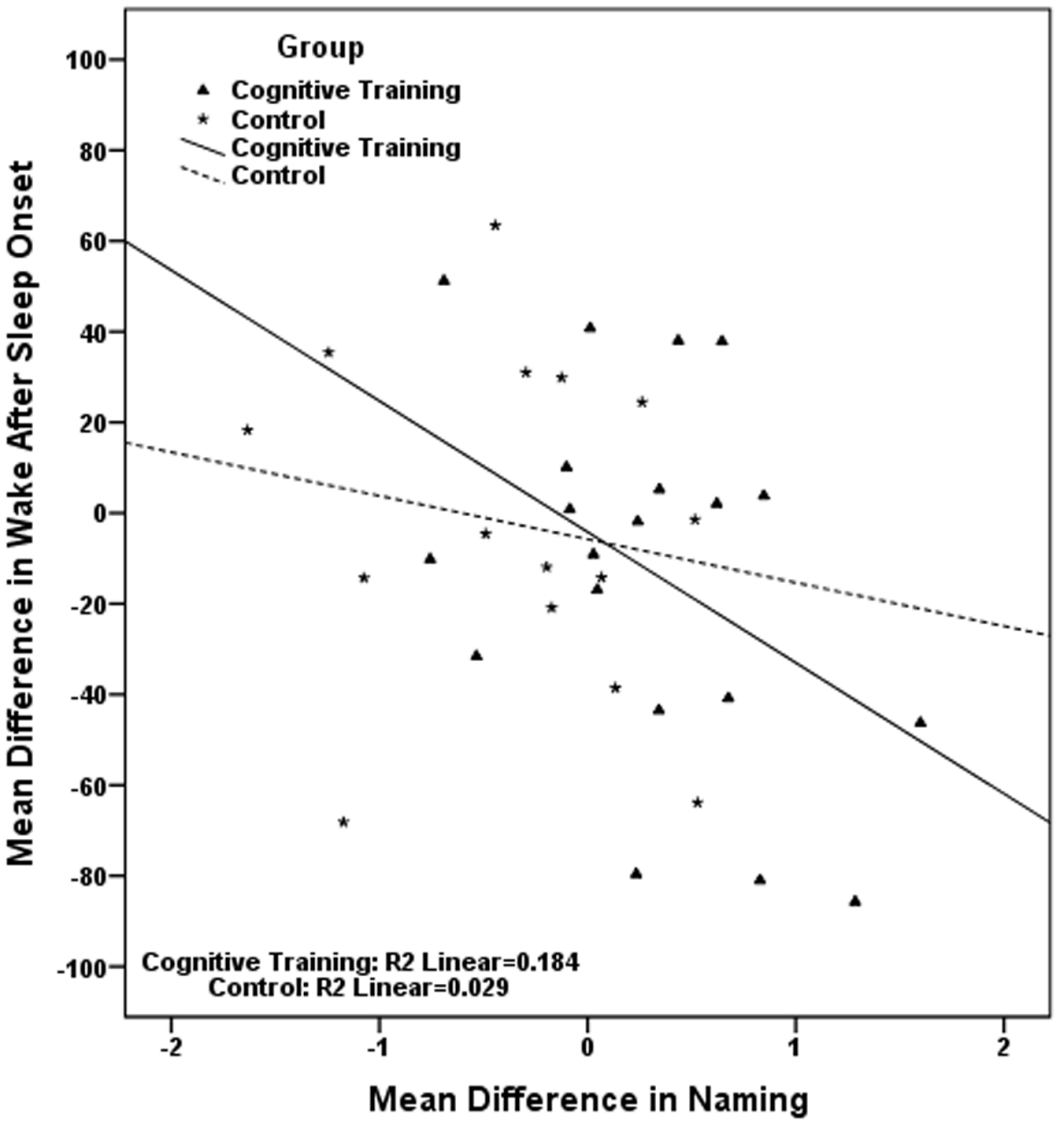
Linear regression between Mean Difference (post-intervention mean minus baseline mean) in Wake after Sleep Onset (dependent) and Mean Difference (post-intervention mean minus baseline mean) in Naming (independent).

**Figure 6:**
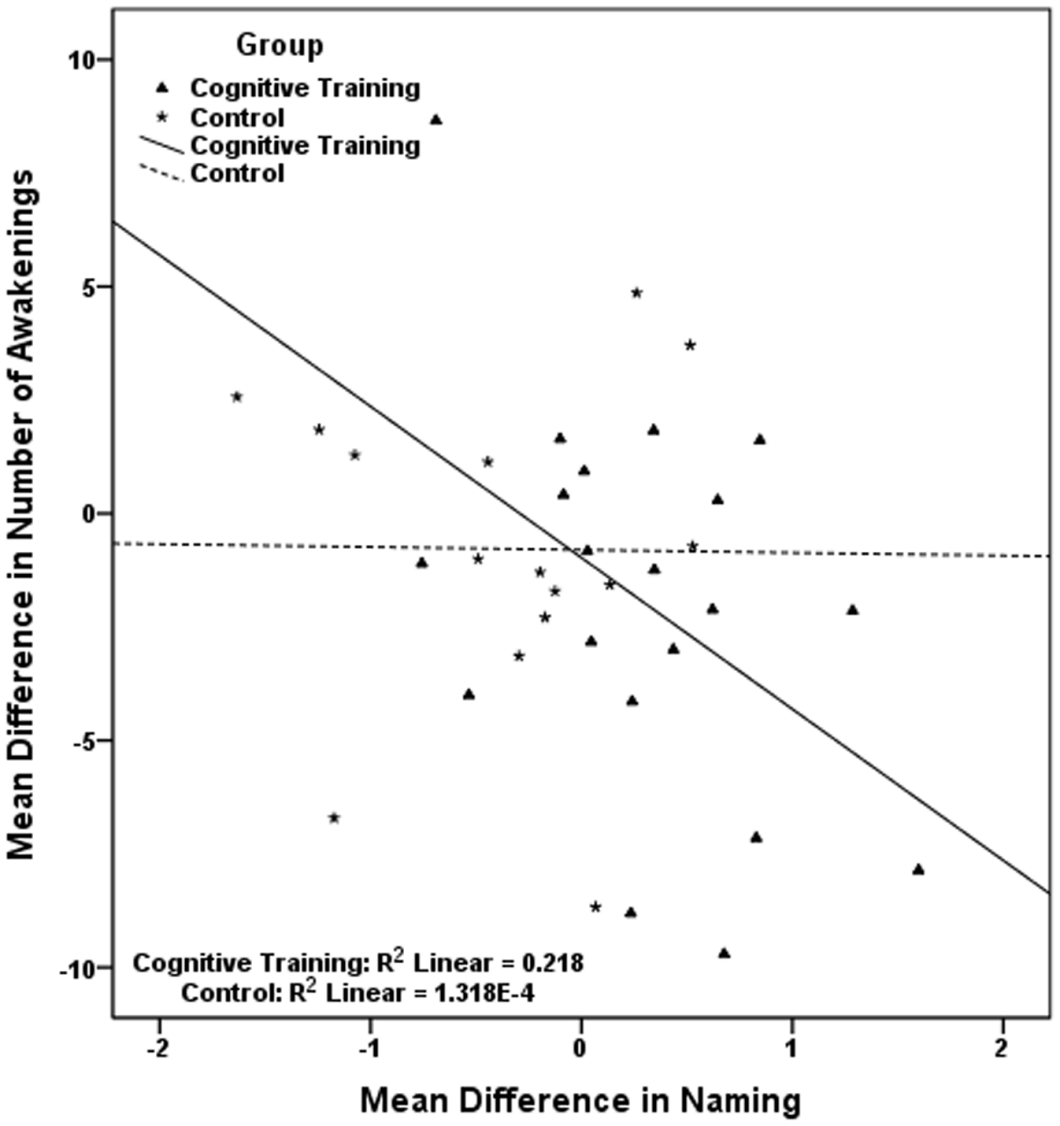
Linear regression between Mean Difference (post-intervention mean minus baseline mean) in Number of Awakening (dependent) and Mean Difference (post-intervention mean minus baseline mean) in Naming (independent).

The pairs of correlated mean differences in Table 6, were used in six sets of hierarchical linear regressions, each set including two different regressions, one for each study group. Results, presented in Table 7, show that in the cognitive training group the improvement in visual scanning is associated with the reduction in sleep onset latency; the improvement in naming is associated with the reduction in wake after sleep onset and with the reduction in number of awakenings, while the improvement in avoiding distracters is related to an increase in total sleep time. Regressions conducted in the control group indicate that the cognitive decline observed in this group in working memory is significantly associated with the longer time required to fall asleep (Table 7). In figures 2, 3, 4, 5, 6 we present the regression lines plotted for these results.

**Table 7:** The prediction of sleep improvements: hierarchical regression results.

Dependent variable	Independent variables	Slope (β)	df	F	adjusted R^2^
Mean-difference sleep onset latency	Mean-difference Working Memory				
Cognitive Training group		−9.28	1,18	1.45	0.03
Control group		−12.10	1,13	8.98*	0.46
**Mean-difference sleep onset latency**	**Mean-difference Visual Scanning**				
Cognitive Training group		8.50	1,18	3.41”	0.11
Control group		4.76	1,13	0.41	0.04
**Mean-difference sleep efficiency**	**Mean-difference Naming**				
Cognitive Training group		5.45	1, 18	2.10	0.06
Control group		3.01	1,13	0.48	0.04
**Mean-difference Total Sleep Time**	**Mean-difference Avoiding Distractions**				
Cognitive Training group		252.33	1,18	5.71*	0.20
Control group		28.66	1,13	0.14	0.06
**Mean-difference Wake After Sleep Onset**	**Mean-difference Naming**				
Cognitive Training group		−28.86	1,18	4.06”	0.14
Control group		−9.58	1,13	0.39	0.05
**Mean-difference Number of Awakenings**	**Mean-difference Naming**				
Cognitive Training group		−3.33	1,18	5.03*	0.18
Control group		−0.06	1,13	0.00	0.08

Significance levels:

“ = significant at the level of.09,

* = significant at the level of.05,

** = significant at the level of.01.

## Discussion

To the best of our knowledge, this is the first prospective study investigating the relation between learning, here operationalized as personalized cognitive training, and sleep quality in older individuals with insomnia. Our results indicate that sleep quality and cognitive function improved as a result of cognitive training for the cognitive training group but not for the Word and Paint control group, and that the improvements in cognitive function predicted the improvements in sleep quality.

The two groups were well matched at baseline, except for a higher mean age in the cognitive training group. However, given that sleep disturbances increase with age, and given that age was controlled for in all analyses, this is unlikely to account for the improvements in sleep parameters observed in the cognitive training group. Other risk factors contributing to cognitive decline or insomnia (age, education, physical and psychological health status) were controlled.

The results of the current study revealed that following cognitive training, sleep onset latency no longer met the criterion for insomnia (>31 minutes) [73]. Sleep efficiency increased, almost reaching the insomnia exclusion criterion (>85%) [74]. In addition, Cohen's *d* obtained for these improvements indicate a higher medium-range clinical significance.

The results of the current study demonstrate that among older adults with insomnia cognitive training improve sleep efficiency. This result can be explained by Fogel & Smith findings [61]. They found that following learning, older adults exhibit an increase in number of minutes in SWS and in SWS percentage [61]. These changes in sleep architecture result in less fragmented sleep and increase the ability to maintain sleep, thereby improving sleep efficiency.

Likewise, our results also revealed that personalized cognitive training in older individuals with insomnia improves avoiding distractions, naming, general memory, visual memory and working memory. This replicated the findings of other cognitive training studies with other populations [38], [38], [41]. For a review of findings in healthy older adults, see Papp et al [94]. A recent investigation of cognitive function in older adults with insomnia found significant differences between participants with insomnia and good sleepers on several aspects of cognitive performance [42], including deficits in memory functioning. The present results suggest that these abilities can be trained and enhanced in older adults with insomnia, and that personalized adaptive cognitive training which targets uniquely impaired functions in each individual may be particularly appropriate for the identification and rehabilitation of these impairments in older individuals with insomnia.

Moreover, our results revealed that in the active control group a significant reduction in mean scores was observed on working memory, a measure which has improved considerably in the cognitive training group. This finding suggests that individuals with insomnia not only experience some difficulty in preserving their existing cognitive status, but in the absence of systematic training which specifically targets well identified deficits, they might experience steep working memory decline.

Whereas change in cognitive function did not, in the active control group, predict improvement in sleep, in the cognitive training group, the associations between improvements in cognitive function and those in sleep quality held both in the correlations and in the regression analysis. The results indicate that improved visual scanning is associated with earlier advent of sleep, while improved naming, a form of declarative memory, is associated with the reduction in wake after sleep onset and with the reduction in number of awakenings. Likewise the results indicate that improved “avoiding distractions” is associated with an increase in the duration of sleep during the night. Moreover, the results indicate that in the active control group cognitive decline observed in working memory is associated with an increase in the time required to fall asleep.

Although causal relationships cannot be inferred from correlation analysis, because the control group experienced no such improvements, it is quite likely that the improvements in cognitive function drove the improvement in sleep quality. The mechanism by which cognitive training may improve sleep is unknown. Here, we propose several possible explanations for this effect. First, sleep and cognitive ability are commonly affected by general ageing processes within the brain, such as atrophy, synaptic degeneration, reduced blood flow and other neurochemical changes [95], [96]. An example for such a type of commonality is the findings of age-related changes of frontal brain activity patterns both during sleep as well as during memory processes [96], [97], [98]. Cognitive training may improve sleep by reducing the impact of these common processes, perhaps through cortical plasticity [95], [99]. Second, cognitive training may improve sleep by changing sleep architecture through an increase in the number and density of sleep spindles, an increase in the duration of Stage 2 sleep, an increase in the duration of REM sleep and REM density, and an increase in SWS sleep. Vertes' view [100] holds that these changes in sleep architecture sub-serve the one principal function of sleep, restitution for the brain, which is achieved through the complementary roles of SWS and REM sleep. In accordance with the “restitution” theory [100]–[102] longer SWS sleep may be required to recover from the mental exertion occasioned by cognitive training, while longer REM sleep may be necessary to periodically activate the brain. SWS sleep is deep and restorative, while REM sleep ensures recovery from sleep by maintaining minimal levels of activity through periodic activation of the brain during sleep [100]. Alternatively, a change in sleep architecture may be occasioned by memory consolidation processes. Studies suggest that sleep facilitates neural activities and interactions taking place in the brain that are thought to promote the consolidation of newly acquired and initially unstable memories [45]–[48]. Therefore, new learning afforded by repeated cognitive training may act as a catalyst to enhance sleep-dependent processes such as memory encoding and consolidation [46], thereby changing the architecture of sleep. A fourth possibility is that cognitive training may have an indirect effect on sleep latency by reducing pre-sleep cognitive arousal, either because subjects were cognitively engaged and had homeostatically “used up” their arousal and/or because cognitive training increased their cognitive fatigue [103]–[105]. The fact that there were no changes in the control group negates the possibility that improvements were due to reducing the opportunities to nap through the activities (increasing the homeostatic drive), but must be related to the cognitive training per se.

The most common treatment today for older adults with insomnia is pharmacotherapy, with a significant number of elderly people taking sleeping pills each day. Yet these medications pose certain risks, such as adverse side-effects and dependence [9], [24], [25], and their effectiveness in insomnia wanes rapidly after 30 days of use [26]. Our findings suggest that for older adults suffering from insomnia, cognitive training should be investigated as a promising non-pharmacological option beneficial in the initiation and maintenance of sleep.

The main limitation of the current investigation was the high attrition rate. Only 51 out of 84 participants (61%) adhered to the training program. Approximately half of the non-completers reported technical problems as their reason for quitting. Technical problems included software crashes and glitches with their home computer that rendered the cognitive training program unavailable or non-functional. Online technology and current versions of the software have since provided answers to such problems.

In addition, 39% of the remaining non-completers reported health problems as their reason for leaving the study. A recent study [39] found that 71% of the subjects in a cognitive training group of multiple sclerosis patients adhered, spontaneously and unprompted, to at least two-thirds of identical cognitive training regimen, and 37% fully completed it. The authors attributed the dropout rate partly to the high levels of fatigue that characterize multiple sclerosis. Since older adults with insomnia also frequently report fatigue [106] this could have been another factor contributing to attrition in the present study.

In summary, the results of the present study suggest that cognitive training may be beneficial in the initiation and maintenance of sleep among older adult insomniacs. However, further investigation should examine the potential long-term improvements and the beneficial effect of combined treatment of cognitive training with cognitive behavioural therapy for insomnia (CBT-I) among older adult insomniacs.

The nature of the relationship between cognitive performance, learning and changes in the structure of sleep or in brain structure warrants further investigation, which not only may shed further light on the relationships between sleep and learning but may also provide important information required to design novel treatments for insomnia among older adults, such as cognitive training.

## Supporting Information

Appendix S1 **Names and descriptions of the training tasks in CogniFit® cognitive training program.**
(DOCX)

Protocol S1 **Trial Protocol.**
(DOC)

Checklist S1 **TREND Checklist.**
(PDF)
