# Supplementary material for: Cognitive Training Improves Sleep Quality and Cognitive Function among Older Adults with Insomnia
Source: PLoS One. 2013 Apr 5;8(4):e61390. doi: 10.1371/journal.pone.0061390 (PMC3618113; doi:10.1371/journal.pone.0061390)
Supplement: Appendix S1 — Names and descriptions of the training tasks in CogniFit® cognitive training program. (DOCX) [file pone.0061390.s001.docx]

*Appendix 1*

Names and descriptions of the training tasks in CogniFit ® cognitive training program

| ***1. The Common Factor***. The name of a semantic category (for example, "transport") is displayed, and various objects are presented either as pictures, spoken words or written words. The user has to decide if each of the objects 'Belongs' or 'Does Not Belong' to the semantic category. This task trains reading-based, visually-based and auditorily-based semantic categorization. |
| --- |
| ***2. A- My Name Is Alice***. An image of an object is displayed on the screen for a short period of time. After the image disappears, four letters are displayed, and the task is to choose the letter, among the four, which is the first letter of the object's name. This task trains naming and working memory. |
| ***3. Up, Down And Around***. Four arrows are displayed in four positions: Up, Down, Left and Right. The users are asked to click on the arrows according to instructions either in auditory or visual instructions modes. Distractions, which the users is asked to ignore, are also embedded in this task. This task trains focused attention, divided attention, sustained attention, shifting and inhibition. |
| ***4. CrissCross***: Two different tasks are given. After training on each of them separately, the screen is divided into two, and one part is activated at a time, displaying one of the tasks. After a few seconds the other side is also activated. The user must switch to the newly activated task as quickly as possible and continue switching each time the other task is activated.  *First task*: A ball is moving toward a wall. If the color of the ball matches the color of the wall it is going to hit, the users must refrain from responding. But, if the ball's color doesn't match the color of the wall towards which it is moving, the users must click on that wall, so the color will change to that of the ball. This task trains focused attention, sustained attention and inhibition.  *Second task*. A ball is slowly moving on the screen. The user is required to track the slowly moving ball by placing the computer mouse cursor on the ball and keeping it precisely at the center of the ball, always moving sat the same speed as the ball. This task trains sustained attention and visual-motor coordination.  When used in concert the two tasks also train, shifting and divided attention. |
| ***5. Chain Code***. A series of digits are presented either in the visual or in the auditory mode. The task is to remember the exact sequence of digits. Both immediate and delayed retrieval are trained in forward and backward sequences. This task trains: delayed memory, visual working memory and auditory working memory in forward and backward sequences. |
| ***6. What Happened To My Mouse***? This task trains and challenges the computer 'mouse' skills. The user is requested to from a puzzle from pieces from puzzle pieces on the screen.As he does so, the mouse begins to behave strangely and move in unexpected directions. The users must continue constructing the puzzle, while, at the same time, controlling the mouse and its dierection as quickly as possible. This task trains visual motor coordination, inhibition. |
| ***7. Pick A Pair***. This is a learning task. The users can choose from three categories: country flags, famous people and monuments around the world. In each of the categories pairs are presented for learning. This task trains visual-verbal paired associate learning. |
| ***8. Music and Rhythm***. In this task, the users first familiarize themselves with the sounds of 10 musical instruments. Later, they are asked to identify the instruments by their sounds, and also to identify some tempo patterns. This task trains visual-musical paired associate learning, musical working memory and divided attention. |
| ***9. Bell Boy***. Two elevators are displayed. The users get a stimulus from each elevator, either visual or auditory. The task is to estimate which stimulus is longer. This task trains time estimation using visual and auditory time-marking stimuli. |
| ***10. Inside And Outside***. A target picture is displayed in the center of the screen. Smaller pictures are also displayed arranged in an outer circle, around the target. The task is to spot the pictures in the outer circle that are similar to the picture in the center. Both the target and the smaller pictures keep changing. This task trains speed of processing, visual scanning, visual perception, inhibition, shifting and divided attention. |
| ***11. Keep The Track***: A route is displayed, with a ball at the starting point. The ball starts moving and the task is to track it with the mouse cursor, always moving at the same speed as the ball. Occasionally the ball and also the route disappear. The task is to continue moving the cursor at the same speed/direction as the ball would be moving had it not disappeared. This task trains sustained attention, fine visual-motor coordination, speed and distance estimation as well as direction. |
| ***12 . Supermind***. The computer chooses a secret code, consisting of sets of two, three or four digits and/or symbols. The task is to break the code by finding out the exact location of each digit or symbol within the set of symbols. This task trains complex problem solving and executive function. |
| ***13. Morning Time***. Windows, arranged at random on the screen, are opened momentarily, one after the other. The task is to follow the exact sequence in which the windows were opened. The task evolves as the user remembers longer sets. This task trains visual-spatial working memory. |
| ***14. Hot Air Balloon***. A hot air balloon flies in the sky. On its way, it lands on different clouds. The task is to remember and reproduce its exact route. This task trains visual-spatial working memory. |
| ***15. Two in One***. Two rooms with different colored walls are displayed. In each room a colored ball is moving on a collision course with a wall. The task is to match the color of the walls to the color of the balls, before the balls collide with the walls, while working simultaneously on the two rooms. This task trains shifting, inhibition, divided attention and response speed. |
| ***1 16. Who And Where***. Pictures are displayed for a short time in random locations on the screen. The task is to remember the exact pictures in their exact locations.  This task trains Visual-spatial processing and visual-spatial working memory. |
| ***17. Hidden Words***. A letter grid appears in the center of the screen. A picture of well-known object appears in the lower left corner of the screen. The task is to  find the name of this object spelled out in the letter grid. This task trains visual search, orthographic structure. |
| ***18. Picture Arrangement***. A segmented/scrambled picture is displayed. The task is to rearrange the picture by moving the pieces to their correct position. This task trains planning. |
| ***19. Picasso***. On the right side of the screen an abstract design is displayed for a short time  only. The left side of the screen displays squares and triangles. The task is to reconstruct the abstract design, using the square and triangles shapes from the left side of the screen. This task trains visual problem solving and visual working memory. |
| ***20. Of Balls And Pictures***. This is a combined task of balls and pictures. First, each task is trained separately and then they are combined so that both tasks can be performed simultaneously. In the ***'Balls'*** task balls are moving on a grid, and the task is to prevent them from colliding. When two balls move towards the same intersection, the task is to click on that intersection, as quickly as possible, before the balls collide. This task trains speed of processing, sustained attention, shifting and inhibition.  In the ***'Pictures' task***, pictures are displayed at the four corners of the screen. The task is to identify similar pictures, and press the 'Space bar'. This task trains: speed of processing, sustained attention, shifting and inhibition.  When the tasks are performed simultaneously, one hand controls the mouse to prevent collisions while the other hand, presses the space bar, each time two identical pictures are identified. Again shifting, inhibition, divided attention, speed of processing are trained but with more complex dual-task situations. |
| ***21. Fast‑Moving Objects****:* A target object appears for a short time on either the left or the right side of the screen and then disappears quickly. The task is to identify the target object from among 4 object options. This task trains visual working memory, visual perception. |
