# Supplementary material for: Cognitive Training Improves Sleep Quality and Cognitive Function among Older Adults with Insomnia
Source: PLoS One. 2013 Apr 5;8(4):e61390. doi: 10.1371/journal.pone.0061390 (PMC3618113; doi:10.1371/journal.pone.0061390)
Supplement: Protocol S1 — Trial Protocol. (DOC) [file pone.0061390.s002.doc]

‏**השפעת אימון קוגנטיבי ממוחשב על איכות שנתם של קשישים אינסומניים.**

**מבוא:**

אחת מהפרעות השינה הנפוצות בגיל המבוגר היא האינסומניה (נדודי-שינה). האינסומניה באה לידי ביטוי במגוון היבטיה של השינה כגון: קושי בהרדמות, יקיצות מרובות במהלך הלילה, עייפות, נמנום במהלך היום ועוד. חומרתה של האינסומניה בגיל המבוגר גבוהה יותר מזו הנצפית בקרב צעירים אינסומנים, כך שמספר ההתעוררויות ואחוז זמן הערנות גבוה יותר במהלך הלילה. כתוצאה מכך איכות חייהם של הקשישים ותפקודיהם היומיומיים נפגעים (Ancoli-Israel, 2004; Ancoli-Israel & Cooke, 2005; Foley, Monjan, Simonsick, Wallace, & Blazer, 1999;).

השפעתה של השינה על יכולת הלמידה, זיכרון, ותפקודים נוירו-התנהגותיים נחקרה רבות. הספרות האקדמאית מצביעה על קשר הדוק ודו כיווני בין שינה ועירות, כך שהשינה עלולה להשפיע על יכולות הלמידה והזיכרון ואילו למידה אינטנסיבית יכולה אף היא להשפיע על דפוס השינה וכן על פעילות מוחית בזמן השינה.

שנת הלילה מורכבת מחמישה שלבי שינה שונים: שלב 1 – שלב 5. שלבי השינה 1 – 4 מכונים שנת NREM, ואילו שלב השינה החמישי, הוא שלב שנת החלום מכונה שנת REM : REM – Rapid Eye Movement.

אחת ההנחות הקיימות והמבוססות היא שהשינה בכלל ושנת ה-REM בפרט, הינה תורם חשוב להבניית הזיכרון (קונסולידציה). במחקרים רבים נחקר תפקידה החשוב של השינה בקונסולידציה תלוית שינה –

Consolidation Sleep-Dependent. על פי תיאורית "הקונסולידציה של הזיכרון בשינה" מידע שנרכש במהלך היום, עובר עירור ומיזוג במהלך השינה, ומגובש בזיכרון לטווח ארוך. תהליך זה מתרחש במיוחד בשלב REM . מחקרים של חסך שנתי הראו כי פגיעה בשנת REM מובילה לירידה בתפקודים קוגניטיביים שונים. מחקרים הדמיה מוחית מצאו כי אזורים נוירו-מוחיים אשר הראו פעילות מוגברת בזמן ערות לנוכח משימה, הציגו רמת פעילות גבוהה ביחס לאזורים מוחיים אחרים גם בזמן השינה שלאחר המשימה (Hornung, Danker-Hopfe, & Heuser, 2005; Steenari, Vuontela, Paavonen, & Carlson, 2003; Walker & Stickgold, 2006).

מטרתו של המחקר הנוכחי היא לבדוק האם ניתן לשפר את איכות שנתם של קשישים אינסומנים באמצעות טיפול קוגניטיבי ממוחשב. בהתבסס על היפותזת הקונסולידציה בשינה אנו משערים כי אימון קוגניטיבי ממוחשב יגביר את הצורך בתהליך גיבוש ומיזוג תכני הזיכרון אשר נרכשו במהלך היום וכתוצאה מכך תיווצר עליה בדרישה לתהליכי קונסולידציה. מאחר ותהליכי הקונסולידציה הינם תלויי שינה ומתרחשים בזמן שנת ה-REM ושנת ה-NREM, אנו משערים כי בעקבות האימון הקוגנטיבי הממוחשב הקשישים האינסומניים ישנו טוב יותר ז"א נצפה לעליה בכמות השינה ושיפור באיכות השינה.

בכדי לוודא שהשינוי באיכות השינה יתרחש הודות לאימון הקוגניטיבי הממוחשב ולא כתוצאה מקיומם של משתנים מתערבים כגון, ריצוי, תשומת לב, שיפור במצב הרוח, חרדה ומשתנים פסיכו-חברתיים נוספים, נבדק במהלך המחקר מדדים אלו לפני האימון ושנית אחרי האימון.

כמו כן על מנת לוודא שהשינוי באיכות השינה יתרחש הודות לאימון הקוגניטיבי הממוחשב ולא כתוצאה מהעבודה מול המחשב לכשעצמה קבוצת הניסוי תתאמן בתוכנת MindFit55, שהינה תוכנה ממוחשבת שפותחה במיוחד עבור קשישים במטרה לאמן את היכולות הקוגנטיביות שלהם, ואילו קבוצת הביקורת תשב מול המחשב פרקי זמן זהים לקבוצת הניסוי אך במקום להתאמן בתוכנה הנבדקים יבצעו משימות פשוטת בתוכנות Word וצייר.

**שיטה:**

נבדקים:

במחקר ישתתפו כ-70 קשישים אינסומניים (על פי מדדי האקטיגרף), בני 65 ומעלה מאזור הצפון והעמקים. לכל הנבדקים יהיה מחשב בבית וידע בסיסי בשימוש במחשב. כמו כן כל הנבדקים יהיו בעלי ראיה ושמיעה תקינה, ישלטו בשפה העברית, לא יינטלו תרופות פסיכוטיות כלשהן ולא יסבלו מהפרעות שינה מאובחנות כגון, דום נשימתי ותנועות גפיים מחזוריות.

כלים ומכשור:

1. טופס הסכמה: במסמך זה יצהיר הנבדק כי השתתפותו במחקר נעשית מרצונו החופשי בלבד וכי הוא מסכים ומודע לכל תנאי הניסוי. הנבדק יודע כי באפשרותו לעזוב את הניסוי בכל עת שיבחר וכי חלה סודיות על נתוניו וביצועיו האישיים ולא יעשה באלו כל שימוש פרט לצורכי המחקר. בנוסף, יצוין כי בתום המחקר יוכל הנבדק להתעדכן בתוצאות המחקר ו לקבל הסבר על מצב שנתו. פרט לכך יינתן מידע אודות הדרך בה ניתן ליצור קשר עם הנסיינים.

2. דף הסבר לנבדק כולל הסבר על שימוש באקטיגרף: הסבר מפורט אודות ההליך הניסויי, שלביו, ואופן השימוש במכשיר האקטיגרף.

3. שאלון דמוגרפי: השאלון כולל שאלות אינפורמטיביות על משתנים דמוגרפים, בריאותיים ותעסוקתיים. כמו כן הוא כולל שאלות לגבי הרגלי השימוש מחשב, הרגלי קריאה, ספורט, מפגשים חברתיים, שימוש בתרופות ועוד.

4. כלי לקביעת מצב מנטאלי: תרגום עברי של שאלון "מיני מנטל" Mini Mental State Examination כפי שהוצג על ידי פולשטיין, פולשטיין ומקהיו Folstein, Folstein, & Mchugh,) (1975. לשאלון בעברית תוקף r = 0.94 בהשוואה למבחן CAMCOG ואלפא קרונבך = 0.82 (Werner, Heinik, Mendel, Reicher, & Bleich, 1999). השאלון מקובל בשימוש שוטף עם אוכלוסיות קשישים כמדד בסיסי לבדיקת מצב מנטאלי ולאישור או שלילת קיומו של תהליך דמנטי. השאלון מורכב משלושים פריטים הכוללים שאלות התמצאות בזמן ובמקום, בדיקת קשב וזיכרון קצר, חישובים פשוטים ומשימות לשוניות וביצועיות. טווח התפקוד הנורמאלי במבחן זה על פי מחקרם של פולשטיין, פולשטיין ומקהיו (Folstein, Folstein, & Mchugh, 1975) מתבטא בציון שבין 26 - 30 (ממוצע – 27.6 וסטיית תקן – 1.7) מתוך 30 נקודות אפשריות.

5. שאלון תרופות: השאלון כלל עשרים ושלושה סוגי תרופות. הנבדק מתבקש לסמן האם הוא משתמש בתרופה מסוימת ובאיזו תדירות. מטרת השאלון היא לאתר נבדקים אשר נוטלים תרופות העלולות להשפיע על תפקודם הקוגניטיבי או על איכות שנתם.

6. שאלון מחלות: השאלון כולל רשימה של עשרים וארבע מחלות לגביהן מתבקש הנבדק לסמן האם הוא סובל ממחלה מסוימת והאם היא מפריעה לשנתו. השאלון מועבר על מנת לאתר נבדקים אשר סובלים ממחלה העלולה להשפיע על תפקודם הקוגניטיבי או על איכות שנתם.

7. סולם דיכאון מקוצר לדירוג עצמי - חובר על ידי(Zung & Durham (1965. סולם זה הוא סקלה לדרוג עצמי אשר נבנתה על מנת לכמת סימפטומים של דיכאון תוך שימוש בקריטריונים דיאגנוסטיים של נוכחות רגש דיכאוני מתמשך ותופעות פיזיולוגיות ופסיכולוגיות מתלוות. המבחן המקוצר מורכב מעשרה פריטים שנלקחו מתוך המבחן הארוך הכולל עשרים פריטים. על הנבדק לדרג כל פריט באחת מארבע האפשרויות: "תמיד", "לעיתים קרובות", "לפעמים", "לעיתים רחוקות או אף פעם". לכל קטגוריה ניתן ניקוד מספרי מ 1 עד 4, כאשר סולם המספרים הצביע על רמת דיכאון הולכת ועולה. לאחר סכימת הנקודות מחולק הסכום בארבעים והתוצאה מוכפלת במאה. סכום נקודות מעל לשבעים מראה כי הנבדק סובל מדיכאון.(Zung & Durham (1965, דיווחו על מהימנות ותוקף גבוהים לסקלה שבנדון. במבחן שנערך לתיקוף הסקלה, נמצאה קורלאציה גבוהה עם סקלה "D" של MMPI ועם הדיאגנוזה הקלינית (r=0.70). כמו כן נמצא שהשאלון מאפשר להבחין בין קבוצות של דיכאונים ואנשים עם תגובות חרדה (P<0.01).

8. שאלון חרדה מקוצר (Anxiety Test) : שאלון זה נבנה על ידי Sinoff, Ore, Zlotogorsky, & Tamir (1999). המבחן המקוצר מכיל עשר שאלות אשר מכמתות סימפטומים של חרדה. על הנבדק לדרג את התשובות באחת מארבע האפשרויות: "תמיד", "לעיתים קרובות", "לפעמים", "לעיתים רחוקות או אף פעם". לכל אחת מהקטגוריות ניתן ניקוד מספרי של 1-4, ככל שהנבדק חרד יותר, עלה סכום הנקודות. סכום הנקודות הכולל שמתקבל מחולק בארבעים ומוכפל במאה. תוצאה של חמישים עד שישים נקודות מראה כי הנבדק גבולי ואילו תוצאה גבוהה משישים נקודות מראה כי הנבדק סובל מחרדה. השאלון תוקף בעברית ונמצא מהימן (אלפא קרונבך = 0.7, r=.73) (Sinoff, et al., 1999)).

9. כלים סובייקטיביים להערכת איכות השינה:

א. שאלון מקוצר להערכת איכות השינה MSQ – Mini Sleep Questionnaire . גרסה עברית שהוצגה על ידי Zomer, Peled, Rubin, & Lavie (1985), כוללת עשר שאלות הניתנות למילוי עצמי. התשובות ניתנות בסולם של 1 – 7, כאשר 1 מייצג "אף פעם" ו – 7 מייצג "תמיד". הנבדק מתבקש לסמן את התשובה המתאימה ביותר לשכיחות התופעה המתוארת בשאלה. חישוב הציון הכללי נעשה על ידי סכימת הספרות שמסמן הנבדק. חישוב הציון בתת סולם אינסומניה נעשה על ידי מיצוע של שלושת הספרות בתת סולם זה (שאלות 1,2,7).

ב. שאלון שינה ארוך: שאלון אינפורמטיבי שנבנה במעבדת השינה בטכניון, ואוסף מידע על הרגלי השינה ואיכות שנתם של הנבדקים במהלך השנים האחרונות. מטרתו לספק מידע מפורט הנוגע להרגלי השינה של הנבדק בכדי לאפיין ביתר דיוק האם ישנן הפרעות שינה כלשהן.

10. כלי אובייקטיבי להערכת איכות השינה: אקטיגרף - מכשיר מדידה אמבולטורי המאפשר מעקב אובייקטיבי אחר מחזורי שינה - ערות. למכשיר האקטיגרף 17 מדדים המעידים על איכות וכמות השינה. בין המדדים המרכזיים: יעילות השינה ( sleep efficiency) לא כולל משך זמן ההירדמות, יעילות השינה כולל משך זמן ההירדמות (sleep percentage), חביון שינה (sleep latency) - מספר דקות עד להירדמות, ממוצע דקות הערות לאחר הירדמות (wake after sleep onset) ואחוז זמן פעילות (activity mean) - המשמש כמדד לחוסר מנוחה (Tanaka et al., 2001). האקטיגרף נענד על פרק כף היד הלא דומיננטית ומכוון למדוד תנועות של שורש כף היד. מכשיר זה הוא מד תאוצה ממוחשב, הדוגם את תנועות היד בקצב קבוע של עשר שניות, בשיטת חציית האפס, כאשר איסוף הנתונים נערך באינטרוול של דקה. באמצעות מחשב ניתן לתכנת את האקטיגרף לקביעת זמן מדויק לתחילת איסוף הנתונים, לקביעת אינטרוול האיסוף ופריקת הנתונים, לצורך עיבוד סטטיסטי. בעזרת אלגוריתם לזיהוי אוטומטי של שינה וערות נערך עיבוד ממוחשב של תאוצות פרק כף היד, המתרגם את הרישום המכאני לדקות של שינה ודקות של ערות. האקטיגרף נמצא מהימן בהשוואה למדידה פוליסומנוגרפית, הנערכת במעבדות השינה ((Sadeh, 1991.

בנוסף, הנבדקים ימלאו "טופס מעקב אקטיגרף" (בוקר / ערב) שיתעד את פעולתם במהלך השבוע בו יענדו את האקטיגרף וכן את הערכתם הסובייקטיבית לאיכות שנתם במהלך שבוע זה.

11. תוכנת MindFit 55 – פותחה על ידי חברת CogniFit, כתוכנה לשיפור הזיכרון והיכולות הקוגניטיביות. התוכנה מותאמת ליכולותיהם הקוגניטיביות של אוכלוסייה המבוגרת (גילאי 55 ומעלה), מאבחנת ומאמנת קשת רחבה של יכולות, תוך שימת דגש על מהירות ודיוק הביצוע. תוכנת MindFit55 מאבחנת ומאמנת 14 יכולות קוגניטיביות עיקריות: קורדינאציית עין- יד; זיכרון וויזואלי קצר מועד; סריקה וויזואלית; חלוקת קשב; זמן תגובה; מודעות; עיכוב (אינהיביציה); הערכת זמן; תפיסה ויזואלית; שיום; תפיסה מרחבית; זיכרון עבודה; תכנון (Planning); וגמישות קשב (Shifting).

התוכנה מחולקת לשלושה שלבים: בראשון נעשית הערכת רמת בסיס, בשלב השני מתבצעת תוכנית האימונים עצמה, ובשלב השלישי מתבצעת הערכה חוזרת.

א. הערכת רמת בסיס (אבחון) – נועדה להעריך את היכולות הקוגניטיביות הנוכחיות של המשתמש, בכדי ליצור תוכנית אימונים המותאמת ליכולותיו של המשתמש עצמו. הערכת בסיס זו אורכת כשעה, ומחולקת לשלוש אפיזודות של כ-20 דקות כל אחת. במהלכה מוצגות 17 משימות קצרות,כאשר כל אחת מתמקדת ביכולת קוגניטיבית עיקרית ובנויה כמעין משחק. בטרם המשימה עצמה מתרחש שלב למידה והתנסות קצר שלאחריו מתחילה המשימה ונמדדים ביצועי המשתמש. פעילותו של המשתמש נרשמת לאורך כל האבחון והמידע המתועד כולל הן את תגובותיו לכל גירוי בנפרד והן חיתוכים סטטיסטים כגון, אחוז התשובות הנכונות ומהירות התגובה הממוצעת לכל משימה ומשימה.

נקודת המוצא בניתוח הביצועים היא ההנחה כי בזמן ביצוע מטלה נמדדת יכולת קוגניטיבית מסוימת ובמקביל פונקציות קוגניטיביות נוספות באות לידי ביטוי. לכן, בעת חישוב ציון כולל של יכולת קוגניטיבית מסוימת, שיטת הניתוח מתחשבת בציונים ממשימות שונות. מתוך שכך, כל יכולת קוגניטיבית יכולה להיות מחושבת על סמך מספר משימות שונות, ונתונים ממטלה אחת יכולים להיכלל בחישוב מספר יכולות קוגניטיביות. בהתבסס על נתוני הנורמות הקיימים וביצועי המשתמש באבחון, ניתן לחשב עבור כל משתמש את הישגיו. בסיום תהליך זה מוצגת בפני המשתמש רשימה של 14 היכולות הקוגניטיביות שנמדדו. בראש הרשימה מוצגות היכולות הקוגניטיביות בהן הוא חזק יותר ואילו בסופה מוצגות היכולות בהן הוא נמצא חלש יותר על פי האבחון שביצע. כמו כן, בשלב זה מונפקת תוכנית אימון המבוססת על ביצועי המשתמש באבחון ומותאמת לו באופן אישי.

ב. תוכנית האימונים – תוכנית האימונים בנויה בהתאם ליכולותיו של השתמש, אשר נמדדו בהערכת רמת הבסיס. תוכנית האימונים כוללת 24 אימונים בני 20 דקות לערך, כאשר כל יום אימון כולל 3 משימות שונות ברמת קושי שונה (קל, בינוי, קשה). תחילת כל משימה מלווה בהסבר הקשור לתוכן המשימה אותה יידרש המשתמש לבצע וכן קיימת האפשרות לצפות במידע אודות ההיבטים הקוגניטיביים העקרים במשימה זו והשלכותיהן על חיי היום יום. בתום ההסבר מתבצעת הדגמה של המשימה על ידי התוכנה ורק לאחר מכן נדרש המשתמש לבצע את המשימה עצמה. בכל שלב יכול המשתמש לחזור ולקבל הסבר נוסף על אופן ביצוע המשימה. בנוסף כל משימה בנויה בדרגת קושי עולה, ומתקדמות בהדרגה, בכדי לבסס את הכישורים הנדרשים לסוג המשימה.

בסופו של יום אימון יכול המשתמש לראות את תוצאות ביצועיו (התוצאות מוסברות למשתמש גם באופן וורבלי), אשר מסבירות את התרומה היחסית של כל משימה למאזן הנקודות. התוצאות מוצגות אף כגרף, המשווה את התוצאות הראשונות (התנסות ראשונית במשימה ספציפית), בנוגע לזמן התגובה ומידת הדיוק עם אלו האחרונות.

ג. הערכה חוזרת – ביצוע חוזר הזהה לחלק ההערכה הראשוני, הנועד לבחון את השיפור

החל ביכולות הקוגניטיביות השונות בהשוואה לרמת הבסיס.

12. חוברת משימות לעבודה ב Word וצייר (Paint) - החוברת פותחה על ידי אחת הסטודנטיות במטרה ליצור תפעול זהה מבחינת כמות וזמני המשימות לאימון של קבוצת הניסוי בתוכנת MindFit. עם זאת, הדגש הושם על בנית משימות אשר לא מערבות פונקציות קוגניטיביות גבוהות ולא בוחנות את זמן התגובה ומידת הדיוק. היכולות הקוגניטיביות מתופעלות ברמה הבסיסית ביותר, ומערבות פעולות שעל פי רוב אוטומטיות עברונו (קריאה, כתיבה). המשימות כוללת הוראות המפרטות צעד אחר צעד מהן הפעולות שעל הנבדק לבצע, בכדי להבטיח שליטה מקסימאלית ואחידות בעבודתם של הנבדקים. חוברות המשימות כללת מטלות המערבות רמת עיבוד נמוכה של: סריקה ויזואלית, תכנון, קורדינאציה – עין יד (שימוש בעכבר), תפיסה חזותית ומרחבית, הוצאת פעולות לפועל, קריאה, כתיבה והבנת הנקרא פשוטה.

13. חוברת הסברים ומידע על תוכנות Word וצייר – החוברת כללה הסבר על מיקומן ואופן השימוש בפונקציות המחשב בהן נעשה שימוש בחוברות המשימות.

הליך:

הליך המחקר יכלול שלושה שלבים עקרים, במהלכם יתקיימו מפגשים פרטניים עם כל נבדק בביתו :

1. העברת שאלונים ומדידת רמת בסיס של איכות השינה – תיערך פגישה בביתו של הנבדק בה יינתן מכשיר האקטיגרף וכן תיקייה אשר תכיל את השאלונים (שאלון דמוגראפי מפורט, שאלון שינה קצר, שאלון שינה ארוך, שאלון הערכת שביעות רצון מהחיים, שאלון להערכת דיכאון בגיל המבוגר, שאלון חרדה, שאלון תרופות ושאלון מחלות). לכל תיקייה יצורפו דפי מעקב עבור ימי מדידת האקטיגרף וכן דפי מידע מפורטים אודות הליך הניסוי ומכשיר האקטיגרף, המציינים באופן בהיר מה הן הדרישות מהמשתתפים בניסוי, ומספרי הטלפון של הנסיינים לשם שאילת שאלות במידת הצורך.

הערכת רמת הבסיס של איכות השינה תתבצע לאורך שבעה לילות.

2. איבחן קוגניטיבי ממוחשב

3. אימון קוגניטיבי ממוחשב בתוכנת MindFit 55 / אימון בתוכנת Word וצייר –

שלב זה בנוי מ – 24 תרגולים מובנים במחשב המתרחשים כ - 20 דקות שלוש פעמים בשבוע, לאורך 10 שבועות.

1. אבחון קוגניטיבי חוזר, הערכה נוספת של איכות השינה ע"י מדדית אקטיגרף במהלך שבוע, וכן העברה חוזרת של כל השאלונים הסובייקטיביים.

**מקורות:**

Ancoli-Israel, S. (2004). A primary care guide to assessing 4 common sleep problems in geriatric patients. *Geriatrics*, *59*, 37-41.

Ancoli-Israel, S., & Cooke, J. R. (2005). Prevalence and comorbidity of insomnia and effect on functioning in the elderly populations. *Journal of the American Geriatrics Society*, *53*, S264-S271.

Foley, D. J., Monjan, A. A., Simonsick, E. M., Wallace, R. B., & Blazer, D. G. (1999). Incidence and remission of insomnia among elderly adults: And epidemiologic study of 6,800 persons over three years. *Sleep*, *22*(Suppl. 2), S366- S372.

Folstein, M. F., Folstein, S. E., & McHugh, P. R. (1975). Mini Mental State: A practical method for grading the cognitive state of patients for the clinician. *Journal of Psychiatric Research*, *12*, 189-198.

Hornung, O. P., Danker-Hopfe, H., & Heuser, I. (2005). Age-related changes in sleep and memory: Commonalities and interrelationships. *Experimental Gerontology*, *40*, 279-285.

Neugarten, B.L., Havighurst, R.J., & Tobin, S.S. (1961). The measurement of life

satisfaction. *Journal of Gerontology, 16,* 134 – 143.

Sadeh, A., Alster, J., Urbach, D., & Lavie, P. (1991). Actigraphically based automatic bedtime sleep-wake scoring: Validity and clinical applications. *Journal of Ambulatory Monitoring, 2*, 209-216.

Sinoff, G., Ore, L., Zlotogorsky, D., & Tamir, A. (1999). Short anxiety screening test-

a brief instrument for detecting anxiety in the elderly. *International Journal of*

*Geriatric Psychiatry, 14*, 1062 -1071.

Steenary, M. R., Vuontela, V., Paavonen, E. P., & Carlson, S. (2003). Working memory and sleep in 6- to 13-year-old schoolchildren. *Journal of the American Academy of Child and Adolescent Psychiatry*, *42*, 85.

Walker, M. P., & Stickgold, R. (2006). Sleep, memory, and plasticity. *Annual Review of Psychology*, *57*, 139-166.

Zomer, J., Peled, R., Rubin, A. H. E., & Lavie, P. (1985). Mini Sleep Questionnaire (MSQ) for screening large populations for EDS complaints. In W. P. Koella, E. Ruther, & H. Schulz (Eds.), *Sleep* 1984 (pp. 467-469). New York: Gustav Fischer Verlag.

Zung, W.W.K., & Durham, N.C. (1965). A self rating depression scale. *Archives of*

*General Psychiatry, 12(1),* 63 – 70.
